# Intimate partner violence and self-esteem

**DOI:** 10.1192/j.eurpsy.2021.2208

**Published:** 2021-08-13

**Authors:** H. Dhouib, S. Omri, M. Daoud, W. Ben Amar, N. Smaoui, R. Feki, M. Maalej Bouali, N. Charfi, M. Maalej

**Affiliations:** 1 Forensic Department, Habib BOURGUIBA University Hospital, sfax, Tunisia; 2 Psychiatry C Department, Hedi chaker University hospital, sfax, Tunisia

**Keywords:** self-esteem, women, assault, Intimate partner violence

## Abstract

**Introduction:**

The impact of intimate partner violence (IPV) can be devastating on women’s psychology. Moreover, IPV may destroy women’s self-esteem and self-identity.

**Objectives:**

To identify sociodemographic characteristics associated with IPV and to assess self-esteem among women victims of IPV.

**Methods:**

It was a descriptive and analytical study over a period of 03 months from June 1^st^ to August 31^st^, 2018 including all cases IPV female victims in forensic department at Habib BOURGUIBA University Hospital, Sfax. In addition to epidemiological data, Rosenberg scale were used to assess the victim’s self-esteem.

**Results:**

Among 142 female IPV victims, only 60 (22.3%) agreed to answer our questionnaire. Their median age was 33.5 years (27-41 years). Victims did not pass high school in 61.7% of cases and they were unemployed in 53.3% of cases. Most women got married at 23 years-old (20-26). The average length of marriage was 7 years (3-14 years). Bruises and abrasions were the most frequent lesions (58.3% and 56.7% of cases). Rosenberg Scale score’s mean was 28.3 ± 4.3. Self-esteem was low or very low among 70% of victims.

**Conclusions:**

Female victims of IPV do not have a specific profile and low self-esteem is quite common among them. Additional research is needed to better understand the extent of the problem and to develop more effective reporting methods.

**Disclosure:**

No significant relationships.

